# An extensive review on phenolic compounds and their potential estrogenic properties on skin physiology

**DOI:** 10.3389/fcell.2023.1305835

**Published:** 2024-01-04

**Authors:** Francesca Rispo, Giulia De Negri Atanasio, Ilaria Demori, Giosuè Costa, Emanuela Marchese, Simón Perera-del-Rosario, Eva Serrano-Candelas, Martina Palomino-Schätzlein, Elisabetta Perata, Federica Robino, Pier Francesco Ferrari, Sara Ferrando, Silvia Letasiova, Jan Markus, Matteo Zanotti-Russo, Elena Grasselli

**Affiliations:** ^1^ Department of Earth, Environment and Life Science, University of Genoa, Genova, Italy; ^2^ Department of Pharmacy, University of Genoa, Genova, Italy; ^3^ Department of Health Sciences, University “Magna Graecia”, Catanzaro, Italy; ^4^ ProtoQSAR SL, Centro Europeo de Empresas Innovadoras (CEEI), Parque Tecnológico de Valencia, Valencia, Spain; ^5^ Departament de Medicina i Ciències de la Vida, Institut de Biologia Evolutiva (CSIC-UPF), Universitat Pompeu Fabra, Barcelona, Spain; ^6^ Moldrug AI Systems S.L, Valencia, Spain; ^7^ Angel Consulting S.a.s 20122 Milano, Milano, Italy; ^8^ Department of Civil, Chemical and Environmental Engineering, University of Genoa, Genova, Italy; ^9^ MatTek In Vitro Life Science Laboratories, Bratislava, Slovakia; ^10^ Interuniversity Center for the Promotion of 3R Principles in Teaching and Research (Centro 3R), Pisa, Italy; ^11^ National Center for the Development of New Technologies in Agriculture (Agritech), Napoli, Italy

**Keywords:** antioxidant, cosmetics, daidzein, endocrine disruptors, estrogen, genistein, polyphenols, skin physiology

## Abstract

Polyphenolic compounds constitute a diverse group of natural components commonly occurring in various plant species, known for their potential to exert both beneficial and detrimental effects. Additionally, these polyphenols have also been implicated as endocrine-disrupting (ED) chemicals, raising concerns about their widespread use in the cosmetics industry. In this comprehensive review, we focus on the body of literature pertaining to the estrogenic properties of ED chemicals, with a particular emphasis on the interaction of isoflavones with estrogen receptors. Within this review, we aim to elucidate the multifaceted roles and effects of polyphenols on the skin, exploring their potential benefits as well as their capacity to act as ED agents. By delving into this intricate subject matter, we intend to provoke thoughtful consideration, effectively opening a Pandora’s box of questions for the reader to ponder. Ultimately, we invite the reader to contemplate whether polyphenols should be regarded as friends or foes in the realm of skincare and endocrine disruption.

## 1 Introduction

Article 2 of both the UK Cosmetics Regulation (EC) No. 1223/2009 (UKCR) and the EU Cosmetic Products Regulation (EC) No. 1223/2009 (CPR) (European Union, 2009) provides a comprehensive definition of a cosmetic product. It encompasses any substance or mixture intended for contact with any of various external parts of the human body, such as the epidermis, hair system, nails, lips, and external genital organs, as well as with the teeth and mucous membranes of the oral cavity. The primary purposes of these products are cleaning, perfuming, altering appearance, correcting body odours, providing protection, or maintaining good condition.

In the present day, the use of cosmetics has witnessed a global surge, becoming indispensable in modern daily routines. Considering our daily routine everyone can count how many cosmetic products are used, from the toothpaste to the shower gel, from a moisturising cream up to a final touch of lipstick or perfume. From hygiene gestures to hydration and protection, from nappy changing to anti-wrinkle face cream; cosmetic products are essential for everyone’s wellbeing, at every age of life. The cosmetics market is worth 10.6 billion (Cosmetica Italia, 2021). Compounds utilized in cosmetics formulations in UK and the EU typically undergo thorough evaluations in compliance with the above-mentioned Cosmetics Products Regulation (1,223/2009). This regulation establishes comprehensive rules for all cosmetic products available in the market to ensure the smooth functioning of the EU and UK market and, most importantly, the safety of such products, ultimately aiming to protect human health at a high level.

In recent times, the cosmetic industry has shown a growing commitment to the production of sustainable cosmetics. In the present scenario, there is significant interest in cosmetics derived from natural resources, which may limit the use of synthetic ingredients. Plants, being a prominent source of biologically active natural compounds with cosmetic and dermatological significance, are gaining attention. In this context, polyphenolic extracts stand out due to their proven antioxidant, anti-inflammatory, anti-aging, antimicrobial, and supportive properties in solar photoprotection (Zillich et al., 2015). On the other hand, polyphenols are also gaining interest in the cosmetics market due to the more attention from the consumer towards environmental preservation (Khan et al., 2019). Furthermore, the polyphenols themselves present antioxidant and anti-inflammatory activities, which are two attractive biological properties in the cosmetic field.

Plant-derived compounds can function as endocrine disruptors (EDs) themselves or become EDs through exposure to various contaminants. In 2002 the World Health Organization (WHO) published the following definition: “*An endocrine disruptor is an exogenous substance or mixture that alters function(s) of the endocrine system and consequently causes adverse health effects in an intact organism, or its progeny, or (sub)populations*” (Bergman et al., 2012). About 5 years ago, EU published a call for data on ingredients with potential endocrine-disrupting properties used in cosmetic products (https://ec.europa.eu/newsroom/growth/items/702447). Group A of this call for data lists “14 substances to be treated with higher priority for assessment as they were undergoing substance evaluation (SEV) under REACH for ED concerns or the SEV had already confirmed ED concerns at the time of publication of the priority list”. The substances under evaluation are: Benzophenone-3, Kojic acid, 4-Methylbenzylidene Camphor (4-MBC), Propylparaben, Triclosan, Resorcinol, Octocrylene, Triclocarban, BHT/Butylated hydroxytoluene, Benzophenone, Homosalate, Benzyl Salicylate, Genistein and Daidzein. It was immediately evident that among these substances synthesized, two are natural polyphenols. In this regard it must be underlined that it is well documented that polyphenols are able to disturb hormone modulation pathways (Cipolletti et al., 2018). Many researchers documented how polyphenols are able to interact with estrogen receptors α (ERα) and β (ERβ). From this point of view polyphenols can be defined as phytoestrogens. In this light some polyphenols are already used as an alternative therapy for menopause (Lephart and Naftolin, 2022).

EU’s call for data on the presence of potential endocrine disruptors (EDs) in cosmetic compounds virtually opened a Pandora’s box, due to the daily and extensive use of cosmetic products. An ED can be found in a cosmetic product due to leaching from packaging or may be present as a contaminant in addition to the active main component, particularly in the case of extraction from contaminated natural products (i.e., contaminated with pesticide or heavy metals). Additionally, the main component itself can act as an ED. Many cosmetic products include polyphenols, which can provide numerous beneficial functions for the skin but may also simultaneously act as EDs.

The EU Commission has undertaken a revision of the Cosmetics Regulation specifically targeting substances that possess ED properties (‘European Commission. Commission Regulation (EU) 2018/605 of 19 April 2018 Amending Annex II to Regulation (EC) No 1107/2009 by Setting out Criteria for the Determination of Endocrine Disrupting Properties. OJ EU L. 101:33–36.’ 2018). It was determined that there are sufficient resources to regulate the utilization of cosmetic ingredients that may pose a potential health risk, particularly those exhibiting ED properties. In terms of environmental considerations, the application of the REACH Regulation is also relevant. The Scientific Committee on Consumer Safety (SCCS) is closely monitoring this procedure and actively involved in evaluating the safety of potential ED substances utilized in cosmetics.

The identification and regulation of EDs aim to protect human health and the environment from potential harmful effects. By understanding and addressing the risks associated with these substances, efforts can be made to minimize exposure and promote safer alternatives in various sectors, including industry, agriculture, cosmetics, and consumer products.

When it comes to polyphenols, which are substances already assumed safely by human beings, the question to ask is whether they are a friend or a foe.

## 2 Polyphenols: a brief overview

**FIGURE 1:**
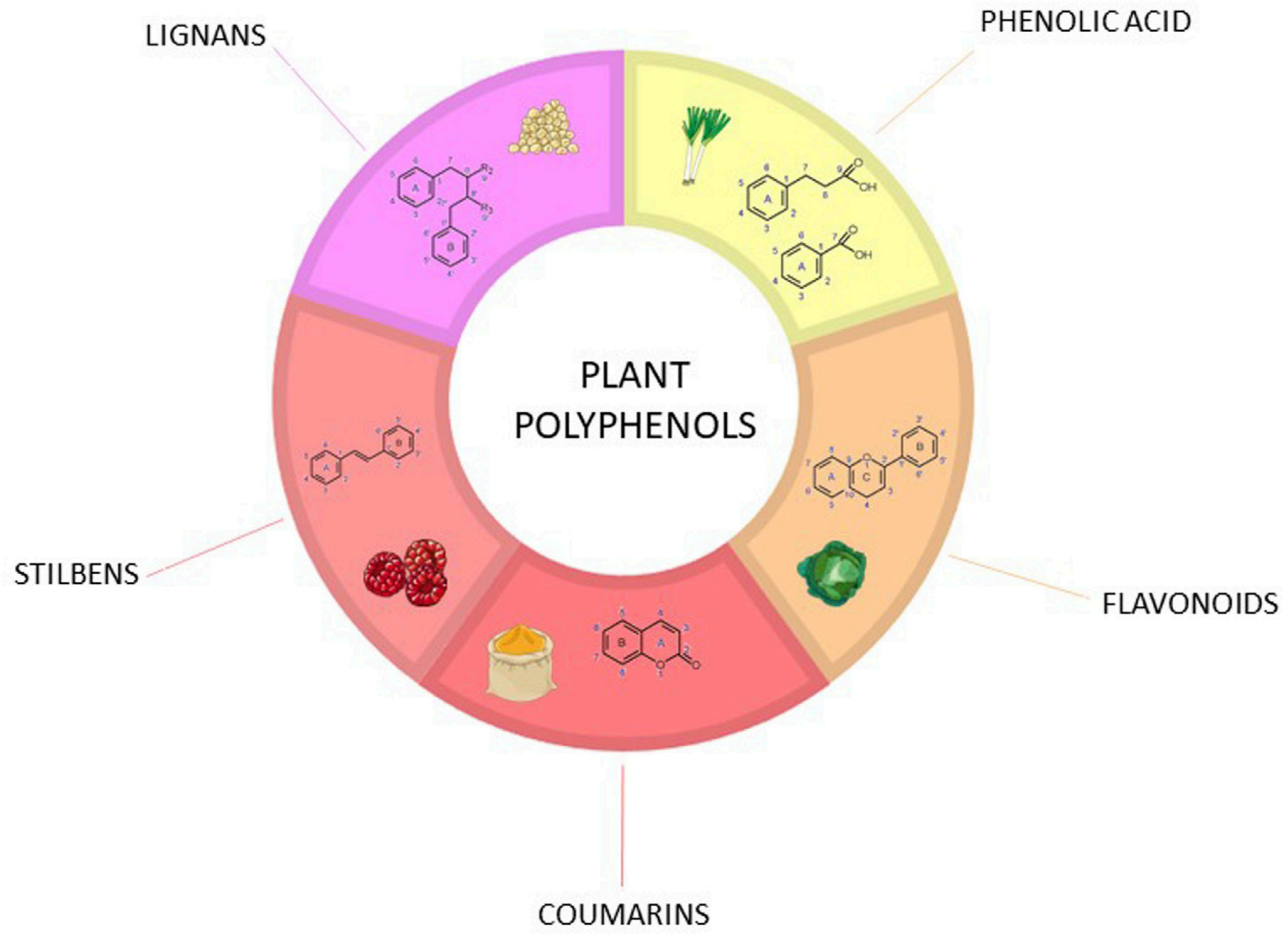
Chemical structure of different groups of polyphenols.

More than 8,000 polyphenolic compounds have been identified in various plant species. Polyphenols may be classified into different groups depending on the number of phenol rings in their structure. In general, polyphenols are characterized by at least two phenyl rings and one or more hydroxyl substituents which can be combined with carbohydrate residues (monosaccharide or polysaccharide) (Singla et al., 2019). The biological properties, bioavailability, antioxidant activity, and specific interactions with cell receptors and enzymes, are related to the chemical structure of polyphenols (Figure 1). It is therefore essential to know the nature of the main polyphenols, their origin, their quantity, their bioavailability, and the factors controlling their bioavailability (Tapiero et al., 2002). Among the different polyphenols, flavonoids are the most widespread group and over 6,000 have been identified (Panche et al., 2016). Flavonoids are structurally derived from benzopyrone displaying a diphenylpropane (C6-C3-C6) basic structure, which possesses two aromatic rings (A and B rings) along with an oxygen-containing heterocyclic ring (C ring) (Bubols et al., 2013). The structural diversity of flavonoid molecules arises from variations in hydroxylation pattern and oxidation state resulting in a wide range of compounds: flavonols, anthocyanidins, anthocyanins, isoflavones, flavones, flavonols, flavanones, and flavanonols (Singla et al., 2019).

Polyphenols are antioxidant compounds capable of scavenging free radicals (Moukette et al., 2015). Antioxidative properties are closely related to the structure of these compounds and result from the dislocation of an electron of the aromatic unit. During the interaction with free radicals, phenoxyl radicals are created and stabilized by the effect of the aromatic ring resonance (Kurek-Górecka et al., 2013).

The main free radicals that exist in our cells and cause oxidative stress are reactive oxygen species (ROS) and reactive nitrogen species (RNS). Endogenous ROS/RNS can be generated as the primary function of an enzyme system (e.g., NADPH oxidases that are activated in response to activated receptors), as a by-product of other biological reactions (e.g., the mitochondrial electron transport chain), or by metal-catalyzed oxidations (e.g., the Fenton reaction: Fe^2+^ + H_2_O_2_ → Fe^3+^_+_ **∙**OH + ^−^OH). The primary ROS/RNS species generated in a cell are superoxide (O_2_^.-^), hydrogen peroxide (H_2_O_2_), and nitric oxide (NO^.^). These molecules can readily react to form other ROS and RNS species. For example, O_2_^**.**-^ can rapidly react with NO^.^ to form peroxynitrite (ONOO−) or dismutate to form H_2_O_2_, and the latter compound can be decomposed through the Fenton reaction leading to the generation of hydroxyl radicals (OH) (Fransen et al., 2012).

Polyphenols can act as antioxidants through several potential mechanisms. The most important mechanism of their antioxidant activity is free radical (ROS and RNS) scavenging, in which polyphenols can interrupt the free radical chain reaction, and suppression of free radical formation through regulation of enzyme activity or chelation of metal ions involved in free radical production (Vladimir-Knežević et al., 2012).

## 3 Beneficial effects of polyphenols: mediterranean diet as a way to prevent a plethora of pathologies

The main route of dietary consumption of polyphenols occurs via ingestion of fruits and vegetables. One of the most widely recognised dietary habits that include the consumption of many polyphenol-rich food is Mediterranean diet (MedDiet). MedDiet is widely employed by the people living in the countries localized in the Mediterranean Sea basin (Finicelli et al., 2019). This diet is characterized by a high intake of polyphenols-rich food such as vegetables, nuts, olive oil, and a moderate consumption of wine, along with rare consumption of red and processed meat, butter, and sugar drinks (Guasch-Ferré et al., 2017). The effect exerted by polyphenols contained within the representative MedDiet components seems to be accountable for the beneficial properties associated with this dietary pattern. Among the healthy dietary patterns, the MedDiet emerges in terms of beneficial properties associated with longevity (Finicelli et al., 2019; Ferrari et al., 2021) since the consumption of polyphenols also improves health status, as indicated by several biomarkers closely associated with cardiovascular risk and metabolic syndrome (MS) (Scalbert et al., 2005). Both men and women who report eating foods closest to the MedDiet were about 10%–20% less likely to die over the course of a study of heart disease, cancer, or any other cause. The longevity of Mediterranean people has been related to olive oil, and its several microcomponents with antioxidant potential, present in all MedDiet variants (Pérez-López et al., 2009). Beneficial effects of the MedDiet on MS and on non-alcoholic fatty liver disease (NAFLD) are now recognized and can be ascribed not only to low calories intake but also to the abundance of antioxidant and anti-inflammatory compounds which are present in fruit and vegetables as well as in herbs and spices widely used to flavour traditional Mediterranean dishes (Baselga-Escudero et al., 2017). Polyphenols are able to alleviate NAFLD symptoms at several levels: i.e., alleviating inflammation, regulating lipid and glucose metabolism by modulating AMP-activated protein kinase (AMPK), increasing adiponectin levels and the expression of its receptors, and lowering the probability of diabetes appearance (Shabalala et al., 2020).

**FIGURE 2:**
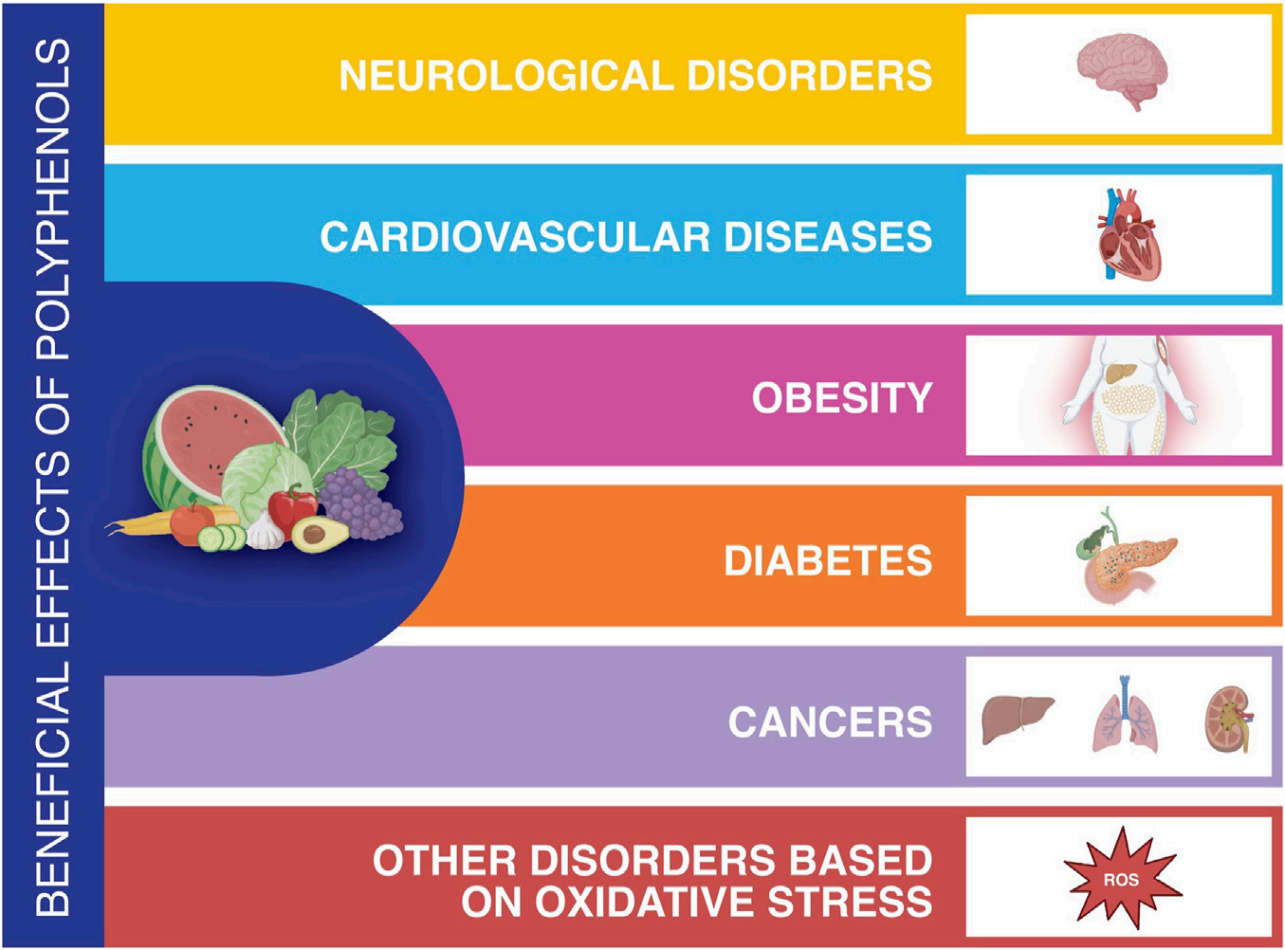
Health benefits of polyphenols related to main diseases (Created using Biorender.com).

In addition to the widely accepted beneficial effects of polyphenols in alleviating NAFLD signs, many other studies have emphasised the beneficial effects of polyphenols regarding their antioxidant, antiproliferative, anti-inflammatory, antimicrobial properties and protection against UV damage (Cutrim and Cortez, 2018) (Figure 2). In most cases, the beneficial effects of polyphenols can be reconducted to a generalised but very effective antioxidant capability. Their antioxidant activity and ability to inhibit enzymes involved in the production of eicosanoids also contribute to their anti-inflammatory properties (Yahfoufi et al., 2018). Eicosanoids, a family of oxygenated metabolites of eicosapolyenoic fatty acids, such as arachidonic acid, are catalysed via the lipoxygenase, cyclooxygenase (COX) and epoxygenase pathways playing an important role in the regulation of several pathophysiological processes, including inflammation and cancer (Agarwal et al., 2009). Several studies have shown that polyphenols such as coffee extracts from Arabica and Robusta cultivars were able to inhibit the proliferation of P388 cells used as a model of leukaemia (LIczbiński and Bukowska, 2022). Thus, polyphenols regulate immunity by interacting with immune cell regulation, proinflammatory cytokine synthesis, and gene expression. Indeed, they inactivate NF-κB (nuclear factor kappa-light-chain-enhancer of activated B cells), modulate mitogen-activated protein Kinase (MAPK) as well as arachidonic acids pathways and can suppress toll-like receptor (TLR) and pro-inflammatory gene expression. Polyphenols also show antibacterial and/or antifungal activity against a large number of bacteria (including Gram-positive and Gram-negative bacteria) and fungi (Manso et al., 2021). Other polyphenols’ targets are inactivating enzymes, binding adhesins on the microbial cell surface, and making microbial substrates unavailable to the microrganisms metabolism (Othman et al., 2019).

Polyphenols also exhibit protective effects against neurodegenerative and cardiovascular diseases (Grosso, 2018) due to their ability to activate several pathways that regulate immunomodulatory and vasodilatory properties (Vergani et al., 2018). These compounds also act in enhancing neuronal plasticity via the CREB (Camp Response Element Binding) protein in the hippocampus by modulating signalling pathways and transcription factors (ERK/Akt) (Carrillo et al., 2019). Furthermore, Leri et al. demonstrated the capacity of olive oil polyphenols (Oleuropein aglycone and hydroxytyrosol) to provide beneficial effects against neuroinflammation and neuronal impairment, both of which are hallmark features of Alzheimer’s disease. The olive oil polyphenols have shown their potential in offering neuroprotection through several mechanisms, including the reduction of pro-inflammatory cytokine release, activation of the TREM2-dependent anti-inflammatory pathway, and the enhancement of protective microglial activity. These actions collectively contribute to the amelioration of Alzheimer’s disease-related symptoms (Leri et al., 2023).

## 4 Possible side effects of polyphenols

The flip side of the coin is that similarly to any chemical substance, polyphenols have the potential to exhibit adverse effects depending on factors such as conditions, dosage, and interactions within the environment (Duda-Chodak and Tarko, 2023). Polyphenols can block iron uptake by chelating the ions of transition metals (e.g., iron and copper) and hinder absorption of iron. While this iron-chelating ability is useful for treating iron overload, it can be detrimental to those with deficiency, potentially causing anemia and interfering with iron homeostasis (Wang et al., 2021). Polyphenols, flavonoids in particular, can interact with proteins both through nonspecific forces (e.g., hydrogen bonding and hydrophobic effects) and by covalent bond formation, forming complexes that impact protein function, solubility, stability, and more. Digestive enzymes, including amylases, proteases, and lipases, can be sequestered by polyphenol binding, thus affecting their activity and potentially nutrient assimilation. This effect can be beneficial, such as in diabetes treatment (Sun et al., 2020; Aryaeian et al., 2017), but detrimental in some other cases, like in iron deficiency or specific intolerances, where enzyme activity disruptions lead to negative consequences. Moreover, in elderly individuals (Rémond et al., 2015) and athletes (Kondo et al., 2019), in which digestive enzymatic function is compromised, polyphenols can hinder digestive enzyme activities, affecting nutrient availability. Polyphenols can affect the digestive system also by altering the intestinal microbiota. The interaction between polyphenols and gut bacteria is intricate, involving the release, conversion, and degradation of polyphenols by bacterial enzymes, influencing metabolites and altering microbial composition. While polyphenols can stimulate or inhibit bacterial growth, dysbiosis caused by their actions might contribute to various health issues, from gastrointestinal disorders to neurological and psychiatric conditions, impacting nutrient availability and metabolic processes (Duda-Chodak, 2012). The interactions of polyphenolic compounds can occur also with drugs and influence drug absorption, distribution, metabolism, and therapeutic outcomes, leading to significant implications for human health. Polyphenols can influence drug-metabolizing enzymes like cytochrome P450, either by inhibiting or inducing their activity, leading to altered drug concentrations and potential risks or reduced efficacy (Mohos et al., 2020). These interactions, which can also affect drug transporters, pose risks for adverse effects, particularly in drugs with narrow therapeutic ranges. Awareness of these interactions is vital for patients and healthcare providers to ensure safe and effective medication use.

Polyphenols, while typically acting as antioxidants, can exhibit prooxidant activity. Under certain conditions (i.e., high level of metals, alkaline pH, and O_2_ presence), polyphenols can display prooxidant behaviour, primarily facilitated by transition metals such as copper and iron. This behaviour arises from the formation of redox complexes involving transition metal ions or phenoxyl radicals (Eghbaliferiz and Iranshahi, 2016). This leads to the generation of ROS, causing DNA damage and lipid peroxidation. Certain polyphenols, particularly those with specific structural features, can induce DNA damage in the presence of copper ions, which might be exploited for targeting cancer cells due to their higher copper content. However, this prooxidant potential warrants careful consideration, as it could harm normal cells and necessitates further research into the conditions that trigger antioxidant-to-prooxidant shifts. In specific scenarios, polyphenols may trigger negative effects on cells, leading to mutagenesis and exerting cancerogenic and genotoxic influences. Research indicates that certain flavonoids, including genistein and EGCG, can induce DNA cleavage through interactions with topoisomerase IIα and IIβ, raising concerns about their impact on cellular processes (Bandele and Neil, 2007). Last but not least, the effect on hormonal imbalance is to be listed and is extensively discussed in this review.

It is noteworthy that most of the *in vitro* studies conducted to prove the harmful effect of polyphenols utilized high doses of polyphenols, which may not be achievable through regular dietary consumption. Consumers often encounter abbreviated positive claims about polyphenols, leading them to opt for supplements containing concentrated polyphenols. However, such supplements can pose risks, containing doses that exceed those in natural foods. While polyphenols are generally safe as part of a balanced diet, excessive consumption through supplements can lead to adverse effects, especially for vulnerable populations. Further research is crucial to understand the conditions under which polyphenols might have negative impacts, particularly regarding their potential to promote cancer. Therefore, it is essential to raise awareness of the potential risks associated with polyphenol supplementation and provide detailed information on their use in various contexts.

## 5 Most common polyphenols and their effect on the skin

Skin aging results from intrinsic and extrinsic factors, leading to reduced skin functionality and structure. UV radiation generates reactive oxygen species (ROS) that damage lipids, proteins, and DNA (M. Sun et al., 2022). ROS-induced DNA damage and the activation of enzymes like collagenase and elastase contribute to skin photoaging, leading to collagen breakdown, elastin abnormalities, wrinkles, and loss of elasticity (Gupta et al., 2023). Plant compounds have shown the ability to counter UVB-induced cellular aging by reducing ROS production in skin cells (Gendrisch et al., 2021).

Beyond their cosmetic effects, the actions of polyphenols on the skin are multifaceted. They encompass anti-allergic effects, protecting skin against solar radiation (Afaq and Katiyar, 2011), antimicrobial properties, and potential for treating skin cancer, preventing photocarcinogenesis, and enhancing hair growth (Kalekhan et al., 2020). These compounds influence immune pathways, microbial membranes, cancer-related processes (Ding et al., 2018) and hair pigmentation (Sun et al., 2022), thereby offering a range of versatile applications as skin therapeutic agents and cosmetic field.

The biological activity of polyphenols in the skin depends on their physicochemical properties and their ability to penetrate the epidermal barrier and reach specific receptors (Ratz-Łyko et al., 2015). Topical products rich in polyphenols can counteract skin changes induced by Sun exposure and aging, repairing damage and enhancing skin thickness. Some polyphenol-rich substances like green tea and grapes show promise against skin aging. However, certain polyphenols can have both protective (antioxidant) and damaging effects (allergic reactions, dermatitis, UV-induced apoptosis) (Korkina et al., 2012).

Green tea polyphenols, particularly (−)-epigallocatechin, (−)-epicatechin, and others, demonstrate protective effects against UV-induced skin damage. These compounds prevent skin damage, lipid peroxidation, and erythema caused by UV exposure. Green tea polyphenols applied topically before Sun exposure can suppress immune responses and inflammation, offering protection against UVB-induced damage (Katiyar et al., 1999).

Tyrosinase, a pivotal enzyme in melanogenesis, produces melanin responsible for skin and hair colour, but its overactivity leads to hyperpigmentation. Conventional inhibitors like hydroquinone can be harmful, urging the search for safer alternatives for sunscreens (Mapunya et al., 2012). Various plant extracts have demonstrated significant anti-tyrosinase effects, highlighting their potential for developing effective and safe tyrosinase inhibitors in sunscreen formulations. Studies reveal promising candidates with strong inhibition, often attributed to antioxidant properties and secondary metabolites (Lourenço-Lopes et al., 2020).

It is important to underline that in addition to polyphenols, other plant derived compounds have the potential to have positive effects on the skin. Oral supplementation with a tomato extract for a period of 3 months has shown significant improvements in the skin barrier. This, in turn, leads to photoprotection and a reduction in signs of aging due to its antioxidant and anti-inflammatory properties (Tarshish and Hermoni, 2023). According to other studies, dietary carotenoids have been shown the capacity to inhibit estrogen signaling of both 17β-estradiol and genistein and attenuate their deleterious effect in hormone-dependent neoplasia (Hirsch et al., 2007). In cosmetic, lycopene is being widely employed formulations because of the protective properties against photodamage and skin aging. It is an excellent tool for preventing skin photodamage because of its capacity to shield cells from oxidative damage (Marchena et al., 2020).

Inflammatory skin diseases cover a wide range of conditions affecting people of all ages and skin types, often exhibiting chronic and relapsing courses. Atopic dermatitis, psoriasis vulgaris, and lichen planus are some examples, characterized by complex causes involving genetics and the environment (Ujiie et al., 2022). Injured stratum corneum releases signaling molecules that trigger cytokine cascades initiating inflammation, contributing to various skin disorders. To manage these conditions, glucocorticoids and biological agents are employed, but systemic corticosteroids and immunosuppressants have short-term use due to serious side effects. Biological therapies target key inflammation pathways, but they also carry side effects like infections and exacerbation of pre-existing conditions (Williams, 2018).

Polyphenols, especially flavonoids, possess potent anti-inflammatory properties and immune regulation abilities (Zeinali et al., 2017). Natural polyphenols like resveratrol, chlorogenic acid, and curcumin modulate pro-inflammatory gene expression and cytokine production, impacting immune cells. Green tea polyphenols and epigallocatechin-3-gallate inhibit prostaglandin metabolites (Chang et al., 2022). Resveratrol induces eNOS, inhibits COX, and inactivates PPARγ. Curcumin downregulates STAT3 and NF-κB and upregulates PPARγ. Caffeic acid phenethyl ester suppresses TLR-4 and NF-κB activation. Quercetin inhibits leukotriene biosynthesis (Yahfoufi et al., 2018).

*Ginkgo biloba* extract promotes repigmentation in vitiligo (Castillo et al., 2023). Carnosol from rosemary reduces inflammation markers in mice. Carnosol also benefits atopic dermatitis lesions (Veenstra et al., 2021; Li et al., 2023). Artichoke polyphenols improve endothelial cell function, skin elasticity, and roughness by acting as antioxidants and anti-inflammatory agents, modulating molecular pathways related to anti-aging mechanisms (Lecci et al., 2021).

## 6 Bioengineered-based strategies for polyphenol encapsulation

Polyphenols hold promise for skin protection and health, but their limited solubility and metabolism challenge their use. Encapsulation techniques and carrier systems offer solutions to enhance their bioavailability.

Polyphenol encapsulation has emerged as an innovative approach within the realms of traditional cosmetics and pharmaceutics, and especially in the innovative sectors of cosmeceuticals and nutraceuticals (Munin and Edwards-Lévy, 2011). Encapsulated polyphenols have the advantage to alleviate their sensory impact, overcome their instability, enhance their skin penetration, and the formulation flexibility of the cosmetic final product. Encapsulation technologies offer a wide range of approaches that aim to protect the inner bioactive molecules from environmental factors (*i*.*e*., light, oxygen, moisture, pH, temperature, and enzymes) thus preserving their biological activity (Paini et al., 2015). These techniques result in a wide spectrum of particles, from nanometric to micrometric ones, depending on the specific process parameters applied during the fabrication procedure. In the context of skin treatment, the most common methods for polyphenols microencapsulation include a combination of spray drying and emulsification (Bucurescu et al., 2018) and complex coacervation (Ang et al., 2019). The possibility to incorporate these microparticles (MPs) in cosmetic formulations in order to establish new innovative products is still an open challenge. Nevertheless, the techniques to produce MPs could have a negative effect on the uniformity of the final product and on its long-term stability. These limitations are due to the disadvantages that are intrinsic in the processes used to obtain MPs. In fact, spray drying is responsible of generating non-uniform capsules and is suitable only for a limited range of coating materials; emulsification is influenced by the toxicity of used organic solvents and droplet agglomeration (Łętocha et al., 2022); and complex coacervation suffers from the fact that the control over encapsulation efficiency and MP morphology remains mainly empirical (Ach et al., 2015). Thus, it is necessary to take into consideration also new emerging technologies focused on the fabrication of nanosized-systems for polyphenol entrapping. These include nanoemulsions, polymeric nanoparticles, and nanoliposomes (Pimentel-Moral et al., 2016), each with its unique advantages and applications. Nanoemulsions, for instance, create nanoscale droplets of polyphenols within a continuous phase, enhancing their stability and solubility (Huang et al., 2010). Nanoparticles, on the other hand, enable precise control over the release of polyphenols, facilitating targeted delivery in cosmetic formulations (Vittala Murthy et al., 2022). Nanoliposomes, consisting of lecithin, water, and cholesterol, are one of the commercialized nanocosmetic products (Ajazzuddin et al., 2015) and they could encapsulate several bioactive compounds.

If, on one side, there are various coating agents that act as good containers for the encapsulation of polyphenols, on the other side, the international scientific scenario is moving towards the use of polyphenols, thanks to their chemical-physical and structural characteristics, as carriers for various biological macromolecules, such as proteins, nucleic acids, and conventional drugs (Wang et al., 2021). This allows to combine the intrinsic properties of polyphenols, *i*.*e*., antioxidant, anti-inflammatory, anticancer, and antibacterial activity (Parisi et al., 2014), with those possessed by the delivered molecules. Examples of these polyphenol-based nanostructures are nanoparticles, phytosomes, and nanoflowers. The drug-phenolic nanoparticles are prepared by co-assembly of phenolic compounds (*e*.*g*., tannic acid, catechin, epigallocatechin gallate, and procyanidin) and traditional drugs (*i*.*e*., bortezomib) (Wang et al., 2018), whereas biomolecule-phenolic nanoparticles consist of polyphenols and biological macromolecules (*i*.*e*., keratin) (Yi et al., 2018).

Another emerging technology is represented by phytosomes, patented by the Italian company Indena S.p.A. (De Negri Atanasio et al., 2021), which are vesicles of phospholipids and phytoconstituents that exert their activities as a consequence of their size, their membrane permeability, and their entrapment efficiency (Tripathy et al., 2013).

Nanoflowers are nanometric structures similar to flowers which have recently attracted the attention of researchers because of their unique properties (*i*.*e*., high porosity, good stability, mechanical strength, cytocompatibility, and high loading capacity) (Mohammad et al., 2022). For skin regeneration, it has been demonstrated that *Aloe vera* gel, known for its emollient properties, can be successfully used to synthesize *A. vera*-incorporated hybrid nanoflowers (Altinkaynak et al., 2023). These emerging nanostructures are addressed mainly to cancer therapy but recently they have started to find an application in skin therapy and regeneration (Durbilmez, et al., 2019; Li et al., 2019).

## 7 Phytoestrogens and their effect on human health

Hormones produced by the endocrine system (Box 1) are molecular signals able to exert a variety of homeostatic actions. Estrogens are a group of steroid hormones that play an important role in the normal sexual and reproductive development in females. They also widely influence many important functions such as cell proliferation and death, liver protein expression, lipid metabolism, energy balance, glucose metabolism, immune and cardiovascular regulation, gonadotrophin feedback and gametogenesis, processing and repair/neurodegeneration, bone growth, and others (Lephart, 2021). 17β-estradiol (E2), the most potent estrogen, delivers its chemical message via ERα and ERβ receptors throughout the body including the skin. The biological effects of polyphenols may extend well beyond the modulation of oxidative stress (Hussain et al., 2016). Polyphenols that are structurally very similar to E2 and are able to bind both ER isoforms are named phytoestrogens (Cipolletti et al., 2018; Gorzkiewicz et al., 2021). These phytoestrogens exert the characteristics of endogenous estrogens (Cos et al., 2003). In this regard, they may act as EDs of the estrogen metabolism (Patisaul, 2017). If the estrogenic effect of phytoestrogens is beneficial or not is still under debate. Most of the scientific literature suggests that dietary polyphenols consumed with fruits and vegetables are safe and their eventual estrogenic action is beneficial for human and animal health. This is likely because of the long-standing co-evolution of both the animal and plant kingdoms. Numerous studies have documented how humans have adapted to the dietary intake of polyphenols at food doses. However, when extracting polyphenols, they may be present in much higher doses than those found in food. In this scenario, humans may not be adapted to such elevated doses, making them susceptible to the detrimental effects of ED activity.

BOX 1 **The Endocrine system.**

The endocrine system is involved in chemical communication to regulate and coordinate nearly every physiological process, including metabolism, reproduction, growth, development, body temperature, blood pressure, and the stress response. The endocrine glands produce hormones, which are chemical messengers released into the bloodstream to trigger appropriate responses in target cells, that can be located at a distance from the endocrine glands (Hiller-Sturmhöfel and Bartke, 1998). In addition to the “classic” endocrine glands, other tissues or organs, such as the gastrointestinal tract, the endothelium, or the skin, can release hormones or their precursors into the bloodstream (Hinson et al., 2010). In response to internal and external stimuli such as those related to toxicity and stress, or as a consequence of experimental conditions, the endocrine activity may appear as either reduced or enhanced (Marty et al., 2018).

A growing number of epidemiological and experimental studies have suggested that consumption of diets rich in phytoestrogens may have protective effects on estrogen-related conditions, such as menopausal symptoms, and on estrogen-related diseases, such as prostate and breast cancer, osteoporosis and cardiovascular disease (CVD) (Lissin and Cooke, 2000). Therefore, phytoestrogens have the potential to regulate all processes that are influenced by estrogen including the induction of sex hormones that bind to globulin and inhibit aromatase (Desmawati and Sulastri, 2019). The interaction of phytoestrogens with ERs also has a positive influence on cognitive function (Soni et al., 2014). Phytoestrogens influence the functioning of the nervous system *via* steroid receptors and 5-hydroxytryptamine receptors or by increasing serotonin reuptake. Phytoestrogens may act as anti-ageing agents in the skin via ERs inducing increasing production of hyaluronic acid, collagen and extracellular protein matrix. In addition, phytoestrogens may also increase skin vascularisation, proliferation, and prevent oxidative stress and apoptosis in the skin (Desmawati and Sulastri, 2019).

On the other hand, consumption and exposure levels are nuanced because adverse effects have been observed at lower concentrations in *in vitro* models (Yu et al., 2021). Studies highlight the ability of genistein to disrupt female reproductive development and function at environmentally relevant doses. Altered estrous cyclicity, altered ovarian function and subfertility/infertility have been shown in female mice exposed perinatally to genistein. Some of these effects may not be apparent until later in life, such as irregular estrous cyclicity, early reproductive senescence and infertility, and therefore would not be detected during the time of exposure (Jefferson et al., 2007). Moreover, some data suggests that *Medicago* and *Trifolium* species, which are used for feeding, can exert detrimental effects on livestock. It is well-documented that certain pasture legume species and varieties can contain phytoestrogens and have the potential to adversely affect ewe fertility. This can lead to a reduction in the lambs born per ewe ratio and/or a decrease in the frequency of multiple births. The detrimental impact stems from the presence of isoflavones within these plant species, particularly *Medicago* and *Trifolium*. Some of these isoflavones have been linked to significant and, at times, lasting negative effects on the fertility of various Australian sheep flocks. This concern highlights the need for attention and further investigation into this matter (Reed, 2016). Another study documented how isoflavone present in the red clover *Trifolium pratense* is responsible for the so-called ‘clover disease’. Since the discovery of clover disease in ewes (Bennetts et al., 1946), concerns have arisen about the consumption of feed containing significant amounts of phytoestrogens leading to potential fertility issues in ruminants. The classical manifestation of clover disease in ewes includes symptoms like infertility, abnormal mammary gland development, lactation issues, and uterine prolapse. Additionally, maternal dystocia can occur due to incomplete dilation of the cervix, particularly when ewes have ingested high levels of isoflavones. Studies have indicated that reduced intake of isoflavones can result in temporary infertility, while prolonged exposure may result in lasting infertility (Marshall, 1973; Adams, 1995; Adams, 1998).

The systemic effects of polyphenols depend on their respective intakes and their bioavailability, which can vary greatly (Manach et al., 2004). Bioavailability and pharmacokinetics of isoflavones are influenced by the texture of food ingredients and the source or form of food consumed. Isoflavones in the form of aglycones are absorbed more quickly than in the form of glucoside conjugates (Desmawati and Sulastri, 2019). The knowledge of dietary intake of polyphenols and their bioaccessibility in the human gut are key factors in assessing their effects on human health (Saura-Calixto et al., 2007). Microbiota can contribute also to the conversion of some polyphenols in more absorbable forms (Davinelli and Scapagnini, 2022). Different microbiota types can be present in human intestine thus leading to the conversion of different polyphenolic compounds and those can be absorbed depending on their solubility (Rocchetti et al., 2022). Absorption, plasma concentration and urinary excretion of phytoestrogens depend on the dose administered as well as the relative bioavailability which, in turn, is influenced by sex (metabolism of phytoestrogens is more efficient in women than in men), intestinal transit time and intestinal flora (Viggiani et al., 2019).

## 8 Estrogenic activity on skin physiology: molecular mechanism of skin ageing

The skin, the largest organ of the human body, constitutes a barrier protecting the body against harmful external factors and xenobiotics. On the other hand, this limits the penetration of active ingredients for cosmetic and dermatological purposes. The skin is made up of three main layers: the hypodermis, the dermis, and the epidermis (Yousef et al., 2023). The hypodermis is the deepest section of skin (made up mainly of fat cells) (Storm-Dickerson and Sigalove, 2017); the epidermis is made up mostly of keratinocytes, rich in ROS, and in low molecular weight antioxidant molecules, and also contains melanocytes, Merkel cells, and Langerhans cells. Instead, the dermis contains most of the connective tissues of the skin, as well as nerve endings, sweat glands, and hair follicles (Menaa et al., 2014).

From a molecular point of view skin is equipped with ERs. ERβ receptors are found on keratinocytes, melanocytes, dendritic cells and vascular endothelium and may affect the proliferation and differentiation of keratinocytes, or inhibit the formation of IL-12 and TNF-α, and regulate the secretion of melanin. ERα receptors are located on fibroblasts and macrophages.

Hormonal signaling is essential for skin physiology. Estrogens dysregulation is associated with modifications such as acne, eczema, and rashes. Thyroid hormones affect epidermal cell functions and cortisol influences metabolism, immune response, and stress management. Hormonal changes due to aging lead to reduced estrogen, progesterone, and androgen levels, contributing to various skin issues. Moreover, functioning as an endocrine organ, the skin itself produces hormones that impact homeostasis and overall health.

As the skin ages, structural changes occur in its synthesis, abundance, and maintenance. Initially thickening up to age 20, the skin progressively thins with age. The epidermis becomes thinner, keratinocytes change shape, and pores increase in size, causing age spots. Collagen, elastin, and hyaluronic acid loss lead to dermal thinning, reduced elasticity, and deepening wrinkles. Collagen fragmentation and oxidative stress play a role. Menopause-related estrogen deficiency correlates with decreased collagen types I and III and skin thickness. Sweat glands distort, blood vessels decrease, and subcutaneous fat distribution changes. These alterations affect the epidermis, dermis, and hypodermis, causing visible signs of aging.

Estrogen administration has positive effects on human skin by delaying or preventing skin aging (Liu et al., 2020). Estrogen effects on skin thickness were first observed in postmenopausal women who had thinner and “transparent” skin due to a decrease in dermal collagen type I. In addition, the decline in skin collagen and skin thickness was correlated to the duration of estrogen deficiency rather than the chronologic age of a postmenopausal woman (Shah and Maibach, 2001). Estradiol administration restores skin thickness by increasing collagen synthesis while limiting excessive collagen degradation. Dryness is also alleviated through increased water-holding capacity, increased sebum production, and improved barrier function of the skin. Furthermore, estrogen modulates local inflammation, granulation and collectively accelerates wound healing (Shu and Maibach, 2011).

Both the quality and quantity of information regarding estrogen and the skin continue to increase as the interests of using estrogen as a therapeutic agent for skin aging, wound healing, and scarring rise. ER activation has been shown to promote wound healing independent of estrogen’s anti-inflammatory properties. Selective estrogen-receptor modulators (SERMs) at ERs have been proven to provide skin benefits. There is tissue-specific expression in humans of the ERs, where ERβ is widely expressed in skin compared with ERα, and this is especially the case in the human scalp. Unlike other nuclear steroid receptors, ERs are able to bind numerous compounds including estrogen analogs, estrogen metabolites, various SERMs, xenoestrogens such as phytoestrogens, marine algae and bacterial compounds, and even some androgen and progestogen compounds (Lephart, 2021).

Different omics analyses have demonstrated age-induced changes in different genes and metabolites related with retinoid acid, a known estrogen-induced metabolite (Everts et al., 2021). In (Kuehne et al., 2017), a decrease on retinoic acid was detected in old human skin in comparison with young skin, which has been related with reduced proliferation and epidermal thinning with age. Furthermore, a meta study including human and mouse samples, showed that seven genes involved in retinoic acid metabolism (including BCO2, RBP1, CRABP2) were altered between old and young skin (Swindell et al., 2012). Both studies suggest estrogen-related retinoid acid changes with age.

## 9 Estrogen-mimicking compounds in cosmetics

Estrogen-deficient skin, especially post-menopause, experiences dryness, wrinkles, impaired wound healing, collagen breakdown, and decrease in barrier function and in antioxidant capacity. The logical healthy approach to addressing skin aging involves exploring estrogens or estrogen-mimicking compounds as potential solutions.

With the rise in interest in long-term postmenopausal skin management, numerous studies on the restorative benefits that estrogen may have on aged skin have expanded. Both ERα and ERβ play a significant role in the skin’s response to estrogenic compounds. ER activation promotes wound healing and other skin benefits.

Exposure to exogenous estrogenic compounds from personal care products, such as phthalates and parabens, raises concerns about their effects on health. Some of these compounds display weak associations with estrogen receptors, and their long-term exposure might lead to estrogenic effects. Parabens, for instance, are commonly used preservatives that have shown estrogenic effects in animal models (Cipolletti et al., 2018). Moreover, cosmetics can include steroidal and non-steroidal estrogens, including phytoestrogens.

The structural similarity to E2 is a distinctive feature for certain polyphenolic compounds from the group of flavonoids occurring in the form of aglycones, especially isoflavones (such as genistein, daidzein, biochanin A, and formononetin) and stilbenes (such as resveratrol). *In vitro* studies on these ligands have demonstrated that they have higher affinity to ERβ than to ERα. Resveratrol exhibits mixed agonist/antagonist activity over the α and β ER. *In vitro* studies for resveratrol, ellagic acid, and 3-()-epigallocatechin gallate have shown the ability to block the ER receptors, leading to antiestrogenic activity, which is associated with cancer chemoprevention (Ratz-Łyko and Arct, 2019).

It has been reported that compounds such as genistein (5,7,4-trihydroxyisoflavone) and daidzein (7,4-dihydroxyisoflavone) possess estrogenic activity. In particular, both compounds have a high affinity for ERβ, and thus may develop estrogen-reactive gene products. The result of this interaction is that it influences the metabolism of steroid hormones or their action, or can modify the structure of ER and ultimately transcription (Rani et al., 2022). Genistein (an aglycone), which is an isoflavone found in high amounts in certain fermented soy foods, has been recently considered an ideal natural selective estrogen receptor modulator (SERM) (Polito et al., 2012). Genistein has been tested in anti-aging cosmetic preparations and has shown attractive results for skin elasticity, photoaging, and skin cancer prevention (Tang et al., 2022). Cosmetic creams containing genistein have been used to improve skin dryness and wrinkles. It was found that genistein increased the thickness of skin collagen by inducing the expression of subcutaneous VEGF (Vascular Endothelial Growth Factor) and increasing TGF-β in the skin (Čoma et al., 2021). Genistein also inhibited MMPs by increasing TIMP protein levels, in turn decreasing the degradation of collagen. Therefore, genistein can significantly increase the thickness of collagen and delay skin aging (Polito et al., 2012).

Daidzein is an isoflavone with extensive nutritious value and is mainly extracted from soy plants (M.-Y. Sun et al., 2016). It has phytoestrogen activity and induces transcriptional changes in extracellular matrix components in dermal fibroblasts (Kim et al., 2014). The estrogenic receptor-dependent transcriptional activity increased in a dose-dependent manner when treated with daidzein, with a maximum of 2.5-fold induction at 10 μg/mL of daidzein compared with the nontreated control. In addition, daidzein significantly increased the expression of collagen type I, collagen type IV, elastin, and fibrillin-1 in human dermal fibroblast (Liu et al., 2020). In cosmetics and personal care products, genistein and daidzein are used as skin conditioning agents. Both ingredients are usually used in leave-on formulations. These are included in the European database for information on cosmetic substances and ingredients (CosIng) with the reported function of ‘skin conditioning’, however additional functions have been reported, including ‘antioxidant’ and ‘skin protecting’.

Resveratrol is now being increasingly emploied in cosmetology and dermatology. This polyphenolic phytoalexin is abundantly present in red grapes and berries has a number of scientifically proven health promoting properties. Its popularity in cosmetology and dermatology is primarily associated with proven ability to penetrate the skin barrier and its antiaging activity. Resveratrol has an affinity for the estrogen protein receptors (both ERα and ERβ), thereby contributing to the stimulation of collagen types I and II production (Ratz-Łyko and Arct, 2019).

The huge involvement of estrogens in the development, progression and treatment of breast cancer raises questions about the potential interactions of chemicals in the environment that may be stored in tissues and that may interfere with the physiological actions of estrogens (Gonzalez et al., 2019). The phytoestrogens can work together with the body’s own estrogens to increase the risk of breast cancer (Stone and Snedeker, 2009). Of particular concern is the possible risk of absorption of chemicals with estrogenic activity or antiestrogenic activity in these products (Myers et al., 2015). An extensive array of cosmetics is applied on and around the human breast on a daily basis, including underarm antiperspirant/deodorant products, body lotions, body sprays, moisturizing creams, breast-firming creams, tanning creams and suncare products. These cosmetics are not rinsed off, as are shampoos or soaps, but the entire application is left on the skin each time, favoring the accumulation of chemicals in the underarm and upper breast area. Such overload of chemicals can result in absorption through the dermis, with the net result of chemical deposition in underlying local tissues. However, in view of the role of estrogens in breast cancer, interference in estrogen action could be a likely effect, and many components of cosmetics have now been shown to possess estrogen-disrupting properties (Darbre, 2006).

Some reports have linked exposure to personal care products and premature female sexual development, as well as gynecomastia in boys and men. If the use of certain personal care products does have significant risks for estrogenic or anti-estrogenic effects, health outcomes of interest would not only include age at menarche and benign gynecologic conditions, but also include menstrual cycle patterns, fertility, and hormonally mediated cancers. Effects in males are also plausible. Perhaps the most vulnerable exposure periods would be during infancy and childhood when natural hormone levels are normally very low. The estrogenic effects of parabens alone are estimated to exceed normal endogenous estrogenic in prepubertal girls (Myers et al., 2015). Parabens (p-hydroxybenzoate esters) are a group of widely used preservatives in topically applied cosmetic and pharmaceutical products (Oishi, 2004). Parabens display weak associations with the ERs *in vitro* or in cell-based models, while they do exhibit estrogenic effects in animal models. Parabens have also been tested as inhibitors of sulfotransferase (SULT) activity in a cellular system, with normal human epidermal keratinocytes. Chronic topical application of parabens may lead to prolonged estrogenic effects in skin as a result of inhibition of estrogen SULTs activity. Accordingly, the skin anti-aging benefits of many topical cosmetics and pharmaceuticals could be derived, in part, from the estrogenicity of parabens (Prusakiewicz et al., 2007).

## 10 How to detect an ED in a cosmetic product?

Due to their unique features hormones and endocrine disruptors cannot be tested as ‘classic’ toxic compounds.

When contrasting the mode of action of an ED in relation to conventional toxicology, it becomes apparent that various factors distinguish their behaviour. These distinctions encompass the dose-response curve trajectory, the multifaceted effects exhibited across different dosage levels, the ramifications over extended periods, the temporal windows of developmental susceptibility, the intricate interplay within mixture effects, and the intricate interactions that may arise in conjunction with other stressors. These intricate dynamics collectively render this class of compounds inherently challenging to evaluate within the confines of traditional toxicological frameworks (Solecki et al., 2017).

Importantly, the evaluation of ED activity through the epidermis necessitates consideration of several salient factors. Chief among these is the intricate physiology of the skin, which functions as a robust barrier against exogenous agents. Consequently, the dose of the ED absorbed through the skin is inherently constrained to be lower than the corresponding intestinal absorption. Then, the resultant systemic exposure, facilitated by the bloodstream, remains comparatively diminished. Notably, the skin stands as an intricate organ harbouring enzymatic machinery capable of metabolizing and thereby modifying putative ED agents. This biochemical interplay within the skin milieu further underscores the nuanced complexity inherent in assessing ED activity. The most intriguing example resides in Somatoline, a medical device containing a synthetic form of the naturally occurring thyroid hormone (T4). Several studies documented that individuals subjected to daily Somatoline administration did not show any signs of toxicity. T4 neither TSH were altered in the serum of these individuals, whereas rT3 (reverse T3) was found to increase. This is due to the conversion of T4 locally in the skin into inactive rT3 by action of D3 deiodinase (Peeters et al., 2003).

Once the quantity of the compound within the circulatory system is established, subsequent investigations encompass the interaction of the compound with plasma proteins, potential hepatic metabolism, and subsequent binding events. In this regard, a pivotal obligatory stride pertains to the potential interaction of the compound with a designated nuclear receptor. In this regard, the CALUX^®^ assays is aimed to detect the endocrine potential of estrogenic compounds through estrogen receptor alpha (ERα) Furthermore, the ERα CALUX assay is standardized since 2018 (ISO 19040-3, 2018) (Wolf et al., 2022).

Debon et al. investigate the estrogenic activity of daidzein and genistein through CALUX assay. They demonstrate that both polyphenols present a significant estrogenic activity, suggesting that both compounds could present an interesting capacity as ingredients for cosmetic formulation inducing the activation estrogen receptors (ERs) (Debon et al., 2023). The ERs have the capacity to bind diverse compounds, typically with two hydroxyl groups separated by a rigid hydrophobic linker, and often featuring a phenolic hydroxyl group (Ascenzi et al., 2006). These structural elements are found in the hormone 17β-estradiol, as well as in the structure of polyphenolic compounds like daidzein and genistein. Molecular docking studies of the human estrogen receptor (ERα) with daidzein and genistein revealed their high theoretical binding affinity for the estrogen receptor (Dhananjaya et al., 2012). In silico simulations were also conducted to assess various analogs of these isoflavones, aiming to enhance binding affinity and identify pharmacophoric groups.

**FIGURE 3:**
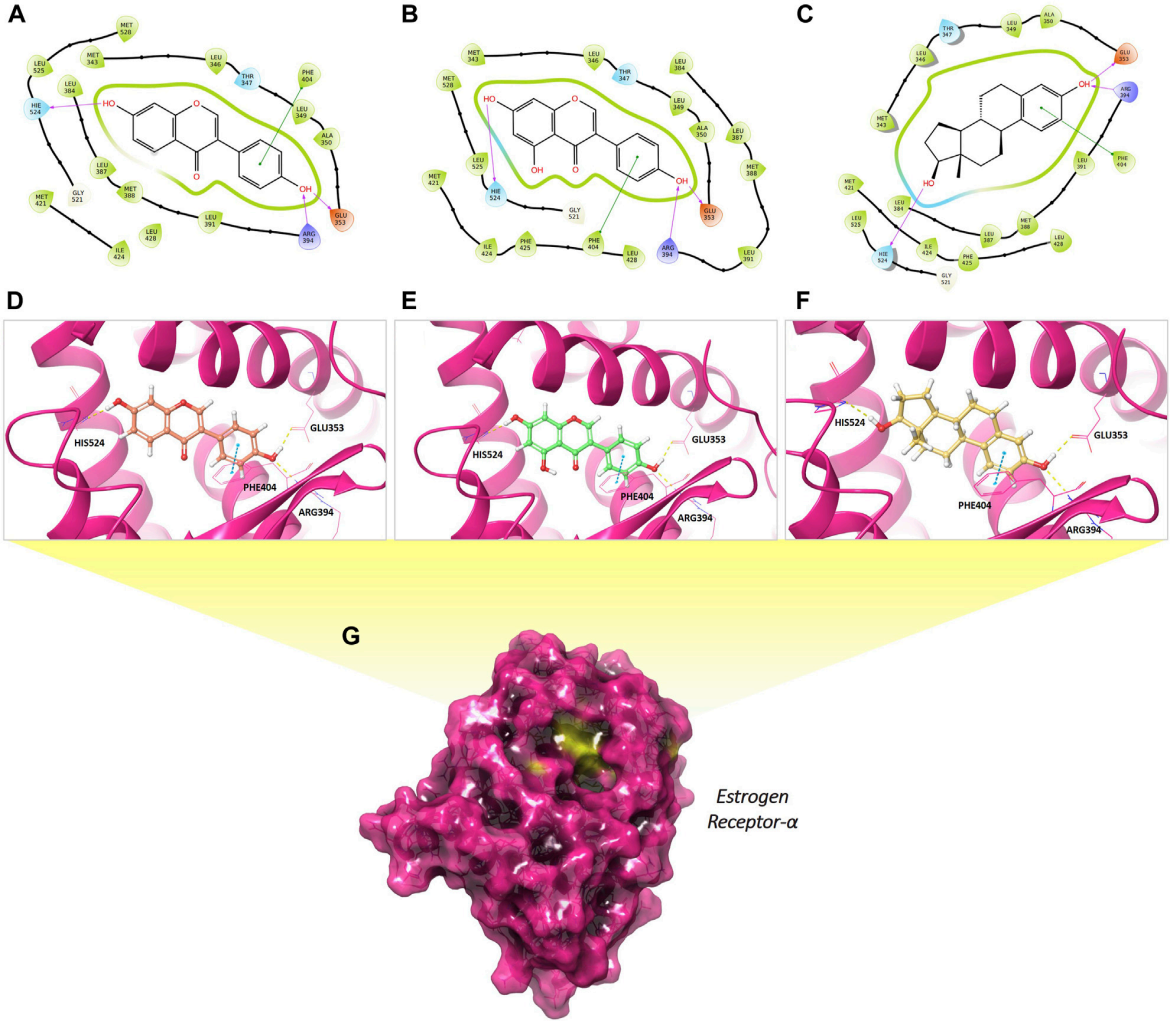
Predicted binding mode of daidzein **(A, D)**, genistein **(B, E)** and 17β-estradiol **(C, F)** in complex with ERα. The 2D representation highlights the key contacting elements of **(A)** daidzein, **(B)** genistein and **(C)** 17β-estradiol into ERα. Panels **(C, D, and E)** illustrate the side chains involved in hydrogen bonds (indicated by yellow dashed line) and π-π interactions (indicated by violet dashed line) between daidzein, genistein, and 17β-estradiol respectively. The surface area of the estrogen receptor-α **(G)** is depicted in solid magenta and the binding site is highlighted in yellow. The graphical representation was generated using the Maestro interface.

In accordance with the findings previously described by Dhananjaya et al., we reproduced the binding mode of daidzein and genistein within ERα, as reported in Figure 3. For this purpose, we conducted molecular modeling analysis by means of the software Glide available in Schrödinger Release 2018-1, (2018), allowing us to elucidate the key interactions between the two natural compounds, the steroid hormone and the ERα binding site. Starting from the X-ray crystallographic structure of ERα, retrieved from the Protein Data Bank (PDB code: 1X7R) (Manas et al., 2004) we demonstrated that the binding mode of the isoflavones, daidzein and genistein, closely mirrors that also predicted for the hormone 17β-estradiol. In all cases, the compounds are well stabilized by hydrogen bonds and numerous hydrophobic contacts within the pocket area of estrogen receptor.

In detail, the phenolic hydroxyl group at position 4’ (4′-OH) was involved in hydrogen bonding network between Glu353 and Arg394 residues, similar to the interaction observed with the 3-OH of estradiol. Another hydrogen bond was established between the 7-OH group of the isoflavone ring system and His524 residue, comparable to that seen for the 17-OH of estradiol. Furthermore, the phenolic ring in all three analysed compounds formed a π-π stacking interaction with Phe404 residue of ERα binding site. Additionally, they were engaged in several hydrophobic interactions with residues Met343, Leu346, Leu349, Ala350, Leu387, Leu391, Met421, Ile424, Leu525, which delineate the binding pocket.

## 11 Discussion

In recent times, the surge in consumer interest towards natural products has been met with heightened awareness. Plants stand as a repository of biologically active natural compounds with significant implications for cosmetics and dermatology. Among these bioactive agents, polyphenols emerge as a reservoir of diverse chemical compositions, offering promising applications. Their antioxidant and anti-inflammatory attributes make them important players in safeguarding skin functions against a range of challenges, spanning from pathogenic assaults to oxidative stress, aging, and even allergies. As such, these compounds have garnered interest as ingredients in various skincare formulations, reflecting their utility in creams and other dermo-cosmetic products. This avenue presents an opportunity to meld cosmetic innovation with sustainable practices.

The consumption of a diet rich in fruits and vegetables, replete with polyphenols, is often associated with a healthy lifestyle. Given their role as estrogen mimics, polyphenols extend manifold advantages, spanning reproductive health, cardiovascular improvements, weight management, cancer mitigation, and more. Pertinently, considering cosmetic application, the primary exposure routes are dermal and inhalation, distinct from the digestive process. Skin’s unique physiology mandates a focused evaluation of substance penetration and the implications for absorption and exposure.

The discourse expands to the potential utility of estrogenic compounds for skin rejuvenation and aging-related concerns. Notably, phytoestrogens have gained traction in anti-aging formulations owing to their potential to mimic estrogenic effects. These compounds, derived from sources such as soy, coffee, olive, and pomegranate, find their place in topical products like creams, serums, and masks, offering an avenue for potential benefits.

The recent call by the EU to assess potential endocrine disrupting properties of cosmetic ingredients underscores the intricate landscape within the cosmetics industry. In a world where cosmetics have become integral to daily routines, the intricate interplay between consumer exposure and endocrine disruption emerges as a challenge of paramount significance.

Cosmetic products, designed to enhance aesthetics and wellbeing, introduce EDs into the equation. These EDs, whether as active agents, additives, contaminants, or leachates from packaging materials, raise the specter of unintended consequences. Given the preponderance of female users, the focus often pivots towards estrogenic interactions and their influence on diverse physiological pathways.

At the forefront of this discourse stand genistein and daidzein, potent phytoestrogens within the polyphenol family. Their scientific exploration is marked by a dichotomy of perspectives, fueling debates over their potential for either benefiting or detrimentally impacting human health. The complexity stems from their dualistic nature, encompassing both health-enhancing attributes and the capacity for adverse effects at higher doses. This ongoing dialogue presents a formidable challenge to the cosmetic industry, navigating the intricacies of incorporating such compounds.

The complexity intensifies when considering polyphenols holistically. Plants, the wellspring of polyphenols, pervade the natural world. This proposition of excluding plant-derived derivatives from cosmetics due to potential endocrine disruption presents a quandary. Paradoxically, these very polyphenols constitute essential components of a healthful diet, an outcome of an evolutionary dance between plants and animals.

The interplay between polyphenols and estrogenic mimicry weaves a nuanced narrative. While these molecules mirror estrogenic effects in the body, bestowing reproductive health, cardiovascular benefits, weight regulation, cancer prevention, and more, their intricate nature defies a binary classification as either purely harmful or beneficial. Contextual factors, such as exposure levels, dosages, and physiological interactions, demand meticulous scrutiny, unveiling layers of complexity.

The discourse shifts to estrogen-deficient skin, an aspect intertwined with aging and menopause. Estrogens, known for enhancing skin parameters like texture, elasticity, and wrinkle reduction, raise inquiries about the feasibility and benefits of incorporating estrogenic compounds into cosmetics, while meticulously managing attendant risks.

Interestingly, parallels can be drawn between cosmetic estrogen exposure and the voluntary consumption of birth control pills, both involving deliberate interaction with exogenous hormones. The differentiating factor lies in the conscious choice, awareness, and informed decision-making associated with birth control pill usage.

In summation, Pandora’s box of naturally-derived endocrine disruptors in cosmetics offers a paradoxical enigma. In traversing this intricate domain, a holistic approach is indispensable, embracing the intricate interplay of evolutionary adaptation, potential health dividends, and associated risks. Striving for balanced cosmetic formulations, informed by the potential benefits of polyphenols while addressing risks, emerges as an imperative endeavor. This multifaceted journey mandates continuous scientific inquiry, ethical contemplation, and rigorous regulatory diligence, harmonizing cosmetic progress with consumer wellbeing.
